# Editorial: Bacteriocins and Other Ribosomally Synthesised and Post-translationally Modified Peptides (RiPPs) as Alternatives to Antibiotics

**DOI:** 10.3389/fmicb.2021.695081

**Published:** 2021-06-08

**Authors:** Harsh Mathur, Des Field, Mathew Upton, Paul D. Cotter

**Affiliations:** ^1^Teagasc Food Research Centre, Co. Cork, Ireland; ^2^School of Microbiology and APC Microbiome Ireland, University College Cork, Cork, Ireland; ^3^School of Biomedical Sciences, University of Plymouth, Plymouth, England, United Kingdom

**Keywords:** bacteriocins, RiPPs, antimicrobials, antimicrobial resistance, genetic engineering

This Research Topic concerns bacteriocins and others RiPPs (Ribosomally synthesized and Post-translationally modified Peptides) as alternatives to antibiotics. Due to the increasing problem of antibiotic resistance globally, there is a pressing need to source such alternative antimicrobials. Here, in this editorial, we summarize the key papers that were published as part of this Research Topic, which consisted of a diverse range of research as well as review articles, pertaining to several different bacteriocins and RiPPs.

A number of these studies report the discovery of novel bacteriocins or RiPPs, either through *in silico* genome mining approaches, laboratory experimental-based approaches or a combination thereof. In one such study, using a combination of lab-based and *in silico* approaches, Angelopoulou et al. reported the discovery of a range of bacteriocins synthesized by strains isolated from human milk. More specifically, the authors isolated bacteriocin producers from 37 human milk samples and found 73 putative bacteriocin gene clusters, which included 16 completely novel prepeptides. Amongst the key findings of the study were the discovery of 2 novel lantibiotics, 3 class IIa bacteriocins, 4 sactibiotics, 1 novel class IIb bacteriocin, 4 new class IIc, and 2 class IId bacteriocins, highlighting that the human milk microbiota is a potentially rich source of diverse antimicrobials. In a separate *in silico* study, Sabino et al. found that ruminal bacteria are a potentially rich source of lasso peptides. More specifically, a genome mining approach was employed by the authors, using tools such as BAGEL4 and antiSMASH5 to screen 425 bacterial genomes from the rumen ecosystem for lasso peptide production. Overall, Sabino et al. found 23 incomplete and 11 complete putative lasso peptide gene clusters amongst the genomes analyzed. Finally, a study by Zendo et al. reported the discovery of a novel nisin variant, kunkecin A, synthesized by *Apilactobacillus kunkeei* FF30-6, a lactic acid bacterium isolated from honey bees. The study describes its antimicrobial activity against *Melissococcus plutonius*, one of the major bacterial pathogens of honeybee broods. Furthermore, the genome sequence of *A. kunkeei* FF30-6 revealed that the biosynthetic machinery of the novel bacteriocin resides in the plasmid pKUNFF30-6. A more in-depth analysis of the kunkecin A gene cluster also revealed that it is quite distinct from the nisin A gene cluster, and is devoid of genes corresponding to *nisR, nisK*, and *nisI*. However, both nisin A and kunkecin A are likely to share similar post-translational modification processes. The structure of kunkecin A was also proposed in the study, on the basis of observed and calculated molecular masses of the peptide. Together, these studies emphasize the growing importance of sequence mining approaches in the discovery of novel RiPPs.

A few studies as part of this Research Topic report the investigation of the activities of nisin variants and/or semi-synthetic hybrids of nisin. In one such interesting study, Reiners et al. provided insights into the antimicrobial activity of nisin H, a natural variant of nisin, in addition to the nisin H F_1_I derivative. The authors expressed the peptide in a heterologous host and determined the yield, the cleavage efficiency using NisP as well as effects on the post-translational modification using mass spectrometry techniques. In addition, the authors determined the effects of nisin H and nisin H F_1_I on the activity of NisI and NisFEG responsible for nisin immunity, as well as the resistance determinants SaNSR and SaNsrFP from *Streptococcus agalactiae*. Overall, the authors found that nisin H and nisin H F_1_I were more potent than nisin A when tested against clinical isolates of *Enterococcus faecalis, Enterococcus faecium* and *Staphylococcus aureus*. In a separate study, Deng et al. reported that biologically active and stable forms of nisin are generated upon conjugation of synthetic hydrophobic polyproline moieties to the lipid II binding fragments of the nisin peptide. The authors used click chemistry technology to conjugate moieties of synthetic polyproline to either nisin AB (encompassing rings A and B of nisin) or to nisin ABC (encompassing rings A, B, and C of nisin). Importantly, the authors found that the conjugation of the synthetic polyproline O63K with nisin ABC resulted in a 16-fold increase in bioactivity, compared to nisin ABC, highlighting the potential of semi-synthetic nisin hybrids as novel antimicrobials. Furthermore, such nisin ABC hydrids are recalcitrant to degradation by nisinase and other proteolytic enzymes at the C-terminus of nisin. These semi-synthetic hybrids, which maintain potent or enhanced antimicrobial activity, could present viable means to circumvent certain nisin resistance mechanisms. Albeit resistance to bacteriocins has been described in several studies over the years, such instances have mainly related to resistance demonstrated in *in vitro* studies rather than in clinical settings/food systems. Thus, bacteriocins may present viable alternatives used in clinical settings, especially in light of the widespread reports of antibiotic resistance globally. In this regard, the use of semisynthetic derivatives of lantibiotics such as nisin and other such hybrid compounds and/or non-ribosomally synthesized peptides, such as those described above, may be a possible means to circumvent issues relating to antibiotic resistance in the clinic. By gaining insights into the precise mode of action of different bacteriocins against specific clinical pathogens, the use of such hydrid compounds could be particularly important as a means to tailor-make narrow spectrum antimicrobials with a view to targeting specific pathogens in the clinic, whilst minimizing the impact on the overall host microbiome.

In relation to class II bacteriocins, a very interesting study by Ross et al. reported the design of synthetic peptide libraries derived from a minimal alpha helical domain of enterocin AS-48 homologs and antimicrobial activities thereof. The alpha helical region of this circular bacteriocin enterocin AS-48 is chiefly responsible for its membrane-permeating and resultant antibacterial activity. The authors conducted homology-based searches of similar domains in several bacterial genomes, used these alpha helical domains as scaffolds to design minimal peptide libraries and assessed their antimicrobial activity. An impressive total of 384 synthetic peptides were evaluated for antimicrobial activity in the study and the authors found that MICs in the low nanomolar range were obtained for the most potent peptides, with no concomitant cytotoxic effects. Thus, studies like this present a blueprint or scaffold for the rational design of semi-synthetic bacteriocin peptide libraries for further evaluation. In a separate study, Vermeulen et al. described the expression of plantaricin 423 and mundticin ST4SA, both class IIa bacteriocins in *E. coli* as a heterologous host, using Green Fluorescent Protein as a fusion partner. Importantly, the authors reported that His-tagged GFP fusion proteins encompassing these bacteriocins, in addition to being autofluorescent, overcame the issue of inclusion body formation, and also decreased the toxicity of these bacteriocins during heterologous expression. The autofluorescence also facilitated real-time quantification of protein yields during heterologous expression.

With regards to studies focused on the potential for bacteriocin resistance development, an interesting study by Campelo et al. reported that a Bce-like bacitracin resistance module in *Lactococcus lactis* is activated upon exposure to the bacteriocin Lcn972. A further possible concern in this regard is that general MFS transporters may contribute to multidrug resistance in pathogenic bacteria, which may also include resistance to bacteriocins. These types of studies are extremely important as they highlight the potential downsides of utilizing bacteriocins, as in certain cases, resistance to other antimicrobials may inadvertently be enhanced as well. YsaDCB is a *L. lactis* ABC transporter that along with TCS-G, forms a detoxification module conferring protection against the cell wall biosynthesis inhibitors bacitracin and the bacteriocin Lcn972. By using a combination of heterologous expression and RT-qPCR, the authors elucidated the precise function of the *ysaDCB* operon. Overall, the authors found that *ysaB* encodes a putative Bce-like permease, whilst *ysaD* encodes a secreted peptide which is likely to be associated with signal relay between the YsaDCB ABC transporter and TCS-G and are together involved in largely similar but somewhat distinct mechanisms of resistance to bacitracin and Lcn972. Telhig et al. published an interesting review article relating to the use of bacteriocins to attenuate antimicrobial resistance in Gram negative bacteria. More specifically, the authors describe the potential use of unmodified and modified microcins, including lasso peptides, nucleotide peptide, siderophore peptides, and linear azole(in)e-containing peptides as a means to decrease the likelihood of antimicrobial resistance amongst Gram negative bacteria. In addition, the review described the potential of the development of cross-resistance and co-resistance in Gram negative bacteria, to various antibiotics and microcins.

Finally, a number of review articles covering a range of topics pertaining to bacteriocins and other RiPPs were published as part of this Research Topic. A very interesting review article by Karbalaei-Heidari and Budisa highlighted the potential of utilizing genetic code expansion strategies in lanthipeptides with a view to attenuating the likelihood of the development of antimicrobial resistance to lanthipeptides. This is because the conventional genetic code and protein engineering strategies are limited to merely 20 canonical amino acids. However, the authors in this review highlight the prospective use of non-canonical amino acids during protein translation with a view to forming semi-synthetic lantibiotics with enhanced biological and chemical properties compared to their natural variants. Another interesting review by Lagedroste et al. summarized recent studies investigating the precise roles and mechanisms of action of enzymes involved in the post-translational modification processes of lanthipeptides. Finally, a review by Rooney et al. summarized the potential of bacteriocins as “plantibiotics of the future” by reporting the details of recent studies of the antimicrobial activity of bacteriocins against Gram-negative phytopathogenic bacteria. It must be noted however that much remains to be elucidated with regards to the impact that extraneous factors such as solar radiation, alterations in light intensity, humidity and temperature have on the stability and antimicrobial activity of bacteriocins and non-ribosomal peptides when potentially being used as antimicrobial agents to control plant infections.

In conclusion, this Research Topic has significantly added to the field of research relating to bacteriocins and RiPPs by highlighting the role of new approaches like *in silico* mining and RiPP engineering, but also addressing some of the potential issues faced by those wishing to commercialize RiPPs for clinical, agricultural, or industrial use.

## Author Contributions

HM wrote the initial draft of the manuscript. DF, MU, and PDC reviewed and revised the drafts. All authors have approved submission of the manuscript.

## Conflict of Interest

The authors declare that the research was conducted in the absence of any commercial or financial relationships that could be construed as a potential conflict of interest.

